# Online health information-seeking behaviour and mental well-being among Finnish higher education students during COVID-19

**DOI:** 10.1093/heapro/daad143

**Published:** 2023-11-07

**Authors:** Hanna Rouvinen, Hannele Turunen, Pirjo Lindfors, Jaana M Kinnunen, Arja Rimpelä, Leena Koivusilta, Markus Kulmala, Kevin Dadaczynski, Orkan Okan, Marjorita Sormunen

**Affiliations:** Department of Nursing Science, Faculty of Health Sciences, University of Eastern Finland, Yliopistonrinne 3, Canthia Building, PO Box 1627, FI-70211 Kuopio, Finland; Department of Nursing Science, Faculty of Health Sciences, University of Eastern Finland, Yliopistonrinne 3, Canthia Building, PO Box 1627, FI-70211 Kuopio, Finland; Wellbeing Services County of North Savo, PO Box 1711, FI-70211 Kuopio, Finland; Faculty of Social Sciences, Unit of Health Sciences, Tampere University, Arvo Ylpön katu 34, FI-33014 Tampere, Finland; Faculty of Social Sciences, Unit of Health Sciences, Tampere University, Arvo Ylpön katu 34, FI-33014 Tampere, Finland; Faculty of Social Sciences, Unit of Health Sciences, Tampere University, Arvo Ylpön katu 34, FI-33014 Tampere, Finland; Department of Adolescent Psychiatry, Tampere University Hospital, Elämänaukio 2, FI-33520 Tampere, Finland; Department of Social Research, Faculty of Social Sciences, University of Turku, FI-20014, Turku, Finland; Research Centre for Health Promotion, Faculty of Sport and Health Sciences, University of Jyväskylä, Keskussairaalankatu 4, Building Liikunta (L), PO Box 35, FI-40014 Jyväskylä, Finland; Department of Health Science, Fulda University of Applied Sciences, Fulda, Germany; Centre for Applied Health Science, Leuphana University, Lueneburg, Germany; Center for Health and Medicine in Society and Center for Health Promotion and Prevention Childhood and Adolescence, Department of Health and Sport Sciences, TUM School of Medicine and Health, Technical University of Munich, Munich, Germany; Institute of Public Health and Clinical Nutrition, Faculty of Health Sciences, University of Eastern Finland, Yliopistonrinne 3, Canthia building, PO Box 1627, FI-70211 Kuopio, Finland

**Keywords:** COVID-19, higher education student, internet, mental well-being, online health information-seeking

## Abstract

Online health information-seeking behaviour has increased since the World Health Organization declared COVID-19 a global pandemic in March 2020. This study examined whether health-related information on COVID-19 searched on the internet was associated with mental well-being among higher education students. A cross-sectional internet survey was conducted among 18- to 34-year-old students in Finland (*N* = 2976; mean age 24.61 years and median 24) in the spring of 2020. The data were analysed using descriptive statistics, Pearson’s chi-square tests, Kruskal–Wallis nonparametric H tests, and a two-way ANOVA. The results indicated that most students (86% of females, 82% of males) used the internet to search for information on COVID-19. Students’ self-perceived abilities to determine the relevance of online information on COVID-19 were associated with mental well-being.

**Contribution to Health Promotion**

- The internet is a popular platform that enables access to health information.
- Many people searched the internet for COVID-19 information during the pandemic. A consideration of the role of the internet in efforts aimed towards disease prevention and health promotion when facing future health challenges is relevant.
- Supporting individual’s ability to understand and use health information retrieved online could contribute to better personal mental well-being.

## BACKGROUND

The internet is a popular source of information and communication because of its easy access and the interactivity it provides. With regard to health matters, it can increase an individual’s knowledge, competence and engagement in promoting, maintaining and restoring health (Wang *et al.*, 2020; Kickbusch *et al.*, 2021). Higher education (HE) students’ internet use for health includes obtaining health information, studying health, communicating about health-related issues and interacting with health professionals online (Rouvinen *et al.*, 2021). The role of the internet as a source of health information among HE students, as well as among the general population, has increased since the World Health Organization (WHO) declared COVID-19 as a global pandemic in March 2020 (WHO, 2020; Schäfer *et al.*, 2021). The pandemic caused isolation, social distancing and lockdowns and highlighted people’s need to find continuing updates regarding COVID-19 and its symptoms (Le *et al.*, 2020). Thus, students sought health information online via social media sites, search engines, dictionaries, specialised health portals, news sites and video platforms (Le *et al.*, 2020; Schäfer *et al.*, 2021). During COVID-19, the most frequently searched information was related to self-diagnosis, self-medication, coping with uncertainty, spread of the coronavirus and current recommendations and restrictions, as well as looking for others with similar health concerns (Zhang *et al.*, 2021; Bak *et al.*, 2022).

A recent concept analysis of health information-seeking behaviour by Zimmermann and Shaw (2020), indicates that because the internet has become a common and preferred channel for retrieving information, there is a need to advance individual’s ability to acquire online health information and use it to make health-related decisions. By the same means, individual ability to determine the relevance of online health information is also needed (van der Vaart and Drossaert, 2017; Palumbo *et al.*, 2022). However, the internet can include inaccurate and poor-quality health information from unreliable sources (Wang *et al.*, 2020), as recognised especially during the COVID-19 pandemic as online health misinformation (Nan *et al.*, 2022). According to a systematic review conducted by Jia *et al.* (2021), online health information-seeking (OHIS) encompasses ‘anything regarding the symptoms, diagnoses, and treatments of different diseases or simply general information about weight loss, healthy diets or wellness tips’. OHIS can include obtaining knowledge for personal health promotion or about health issues or problems and information to support health decisions or behaviour changes on a daily basis (Wang *et al.*, 2020; Jia *et al.*, 2021). Thus, health information-seeking behaviour includes active or purposeful behaviour undertaken by an individual with the objective of finding information about health (Zimmermann and Shaw, 2020). In 2020, in Finland, 77% of people aged 16–74 years searched online for health-related topics, placing Finnish people as the top active online health information seekers in the European Union (Eurostat, 2021). According to a review and a meta-analysis, the universal or dominant predictors for OHIS remain uncertain (Wang *et al.*, 2020). Previous evidence indicates that different factors, such as age and gender, may influence OHIS. For instance, females are more likely to seek health information compared with males, and an older age is associated with decreased levels of motivation for OHIS (Baumann *et al.*, 2017; Nikoloudakis *et al.*, 2018).

In understanding the mechanisms of HE students’ intentions to seek COVID-19-related information, the Planned Risk Information Seeking Model (PRISM), developed by Kahlor (2010), can be used. The PRISM model incorporates aspects of several models used in health communication, such as the Theory of Planned Behaviour (Ajzen, 1991), the Risk Information Seeking and Processing Model (Griffin *et al.*, 1999; Kahlor, 2010) and the Comprehensive Model of Information Seeking (Johnsson and Meischke, 1993). According to the PRISM model, several individual-level factors shape information-seeking intentions: subjective seeking-related norms (social pressure to seek information), attitudes towards seeking information (individuals’ evaluations of the information-seeking behaviour), perceived knowledge (individuals’ perceived knowledge about health risk), perceived knowledge insufficiency (that an individual has insufficient knowledge about a risk), risk perception (individuals’ perceived susceptibility to the health risk and the perceived severity of the illness) and affective response to risk (worry or anxiety; Kahlor, 2010; Wang *et al.*, 2020).

Research has shown diverse evidence regarding the relation between health information-seeking behaviour and well-being among HE students during the COVID-19 pandemic (Capone *et al.*, 2020; Schäfer *et al.*, 2021). Students with a high frequency of searching for information reported higher levels of positive well-being and risk perception (Capone *et al.*, 2020), whereas students who reported a heavy reliance on online information demonstrated a high risk of health mismanagement (Zhang *et al.*, 2021). The online sources used are also linked to well-being. For instance, frequent exposure to social media has been associated with a high prevalence of mental health problems (Gao *et al.*, 2020), and proper use of social media for information purposes has been perceived as beneficial in improving psychobehavioural responses to COVID-19, for example, in areas of higher perceptions of severity, self-efficacy and perceived control or intention to carry out prevention measures (Lin *et al.*, 2020).

During the COVID-19 pandemic, health information was mostly available online, making digital health literacy (DHL) an important concept (Htay *et al.*, 2022). DHL consists of the skills of searching, selecting, appraising and applying online health information (van der Vaart and Drossaert, 2017). Health literacy has been defined in several ways. It is foremost perceived as an individual ability to obtain and translate knowledge and information in order to maintain and improve one’s health in a way that is appropriate to the individual (Liu *et al.*, 2020). Furthermore, health literacy comprises different skills, and it varies from individual to individual. It is noteworthy, however, that someone with a high level of health literacy skills may experience challenges in applying them (Nutbeam and Lloyd, 2021.) For example, an individual may experience OHIS as easy but end up reading information that is consistent with the person’s existing beliefs and thus ignore inconsistent, although relevant, information (Meppelink *et al.*, 2019). During the pandemic, the individual ability to integrate information into behavioural actions was a challenge (Bak *et al.*, 2022). Among students, this might be because of a fear of the inadequacy and the rapidly changing nature of health information found online (Patil *et al.*, 2021). Information available online is not formally controlled, and therefore its trustworthiness varies (Meppelink *et al.*, 2019). Hence, the individual ability to use the internet to access, collect, understand and process health-related information is essential (van der Vaart and Drossaert, 2017; Palumbo *et al.*, 2022). OHIS behaviour requires health literacy skills that will help people guide their health-related decision-making and behaviour (Zimmermann and Shaw, 2020; Schäfer *et al.*, 2021).

Health and well-being are multidimensional concepts with contributions emerging from various disciplines and contexts (Hernández-Torrano *et al.*, 2020). In the focus of this study, the definition of mental well-being refers to ‘a state in which individual realises his or her own potential, can cope with the normal stresses of life, can work productively and fruitfully, and is able to make a contribution to her or his community’ (WHO, 2018). Studies of mental well-being have highlighted that demographic factors, such as age and gender—older age and female gender, specifically—influence well-being (Huang *et al.*, 2018). Among students, studies have addressed an increase in the number and severity of mental problems and help-seeking behaviours, indicating a strain on students’ mental well-being (Kalkbrenner, 2016; Lattie *et al.*, 2019). Moreover, current studies demonstrate that students’ well-being is affected by the COVID-19 pandemic and the social restrictions caused by it. In particular, symptoms of anxiety, posttraumatic stress and depression have increased since the onset of the pandemic (Sarasjärvi *et al.*, 2022). These findings indicate that there is an urgent need to further assess the mental well-being and OHIS behaviour of HE students during these exceptional times (Capone *et al.*, 2020; Schäfer *et al.*, 2021).

This study examines OHIS behaviour during COVID-19 among Finnish HE students and its association with their mental well-being; specifically, we focused on the following questions:

1) How common is online information seeking for COVID-19 among HE students, and does it vary by gender and age?
2) How do HE students determine the relevance of online COVID-19 information to everyday health decision-making? Do the self-perceived abilities of applicability and usability vary by gender and age?
3) Is COVID-19 online information-seeking associated with HE students’ mental well-being and, if so, does this association vary by gender and age?
4) Is the ability to determine the relevance of online information about COVID-19 associated with HE students’ mental well-being? Does this association vary by gender and age?

## METHODS

### Design

This cross-sectional web-based survey among Finnish HE students was carried out in the beginning of May 2020, 2 months after the lockdown of HE institutions in Finland. The study was a part of the larger international COVID-19 Health Literacy Research Network, which includes more than 70 countries worldwide (COVID-HL Network, 2023) and was approved by the Bielefeld University Ethics Committee (No. EUB 2020-053). Permission for data collection was obtained from each of the six HE institutions involved in the study in Finland. The ethical guidelines of the Finnish National Board on Research Integrity TENK (2022) were followed and are available at: https://tenk.fi/en.

HE in Finland is carried out by a dualistic model: by (1) academic universities (UNIs) conducting scientific research and providing undergraduate and postgraduate academic education and (2) universities of applied sciences (UASs), which provide professional, work-related education and applied research and development (Ministry of Education and Culture, 2023). Four national UNIs and two UASs participated in this study. HE institutions’ study services, student unions and associations were contacted by email to reach out to the students. Participation in the study was voluntary, and students gave their active consent to participate. The survey was administered in an electronic form using the online statistical survey web app LimeSurvey (LimeSurvey GmbH, 2021).

### Measures

Sociodemographic information included age and gender. Age was measured in number of years and gender was asked as female, male or diverse.

#### Online health information-seeking (OHIS)

OHIS on COVID-19 was assessed using the following question: ‘Have you searched the internet in the last four weeks for information about the coronavirus? This may include, for example, information of infected cases, or avoiding or dealing with restrictions in everyday life’. The response options were: ‘Yes, only information for me’; ‘Yes, only information for other people’; ‘Yes, information for me and for other people’ and ‘No, I haven’t searched for information in the last four weeks’. For the analyses, these responses were dichotomised into ‘Yes’, combining the three yes answer options and ‘No’.

#### Determining the relevance of health information

Students’ self-perceived ability to determine the relevance of COVID-19-related online information for daily decision-making and for health was surveyed using an adapted version of one subscale (determining relevance) of the DHL Instrument (DHLI; van der Vaart and Drossaert, 2017). The DHLI is theory based and has been tested empirically, referring to self-perceived skills needed for health-related internet use, including finding and appraising online health information as well as communicating via social media and protecting one’s privacy. The subscale used to determine the relevance of health information included three items that were adapted to the context of COVID-19 (Dadaczynski *et al.*, 2020). Students were asked how difficult or easy it is to (i) decide whether the online information found is applicable to their decision-making, (ii) apply the online information they found to daily life and (iii) use the online information they found to make decisions about their health (e.g. in regard to protective measures, hygiene regulations, transmission routes and risks and prevention). The level of ease was measured using a 4-point Likert scale with response options being 1 = very easy, 2 = easy, 3 = difficult and 4 = very difficult. The determining relevance subscale was chosen because the above-mentioned single items focus on the individual’s ability, and the focus of this study was on examining the individual’s perspective. Health information-seeking behaviour is driven by the individual’s information needs and the ability to find, comprehend and use information one finds when making informed health decisions (Wang *et al.*, 2020; Zimmermann and Shaw, 2020; Jia *et al.*, 2021).

#### Mental well-being

Mental well-being was examined using the WHO Well-Being Index (WHO-5; WHO, 1998), a short subjective measure of current mental health. The WHO-5 has been found to have a good-construct validity as a unidimensional scale measuring well-being (Topp *et al.*, 2015). It consists of five items in relation to the past two weeks: (i) ‘I have felt cheerful and in good spirits’; (ii) ‘I have felt calm and relaxed’; (iii) ‘I have felt active and vigorous’; (iv) ‘I woke up feeling fresh and rested’ and (v) ‘My daily life has been filled with things that interest me’. The items are rated on a 6-point Likert scale, ranging from 0 (*at no time*) to 5 (*all the time*) and multiplied by 4. This results in a mental well-being sum score ranging from 0 to 100, with higher values indicating a higher well-being. A score ≤ 50 indicates a low mental well-being, and values ≤ 28 indicate a need for further diagnostic clarification because there is a higher probability of depression (WHO, 1998; Topp *et al.*, 2015). The WHO-5 was chosen because it is a widely used questionnaire in the assessment of subjective mental well-being in research and clinical settings (Topp *et al.*, 2015).

### Data analysis

Participants’ age (18–34 years) was divided into four categories (< 22 years, 22–24 years, 25–29 years and 30–34 years). Respondents who reported their gender as female or male were included in the analyses because the number of respondents who reported their gender as diverse was small (2%, *n* = 61). A chi-square (χ^2^) test was used to test the difference in searching or not searching health-related information between gender and age groups. Cramér’s *V* effect size measurement was applied for the chi-square test of independence. This is a measure of the strength of the association between two nominal variables ranging from zero to one (0 indicates no association between two variables, 1 indicates a complete association between the two variables; Cramér, 1946). A nonparametric Kruskal–Wallis test was used for testing the difference between genders and age groups in students’ self-perceived ability to determine relevance of online information found about COVID-19 and the related issues for daily decision-making and for mental health. For the determining relevance subscale, the rating scale questions were transformed into numbers ranging from one (*very easy*) to four (*very difficult*). In addition, an extra analysis of the dimension of the single three items of the determining relevance subscale was carried out. First, sum variables were created from the items, and the differences between the sum variables were examined with the Welch’s Two-Sample *t*-test and an analysis of variance (ANOVA) to determine whether there was a significant difference between the means. A Cronbach’s alpha coefficient was used to calculate the internal consistency coefficients of the items included in the subscale. The alpha was good (0.81), given that the acceptable level is 0.70 or above on a scale from zero to one (Cronbach, 1951). ANOVA was used to test the differences in the WHO-5 mental well-being index among all different explanatory variables (age, gender and self-perceived ability to determine the relevance of online information on COVID-19). Statistical significance was set at *p* < 0.05. R statistical software was used to conduct the analyses (R Core Team, 2022).

## RESULTS

The sample consisted of HE students (*N* = 2976) age 18–34 years. Of the participants, 74% were females (*N* = 2208) and 26% were males (*N* = 768).

For the interpretation of mental well-being scores, a cut-off score of < 50 is considered as indicating low well-being, and 100 denotes the highest possible well-being. The mental well-being scores stratified by gender were, for females, as follows: first quartile (Q1) = 40, median (*Mdn* = 52, mean (*M* = 52.48, Q3 = 68). For males, they were as follows: Q1 = 44, *Mdn *= 56, *M = *54.74, Q3 = 68. The scores were quite congruent between the genders.

### COVID-19 information sought from the internet

The majority of the students (85%) searched the internet for COVID-19 information. A statistically significant gender difference could be identified, with a higher percentage of females (86%) reporting searching for information compared with males (82%; *p *= 0.018, Cramér’s V effect size = 0.04: Supplementary File S1). In addition, with increasing age, a higher percentage of students reported searching for online information on COVID-19 (*p *= 0.004, effect size coefficient = 0.06).

### Ability to determine the relevance of the COVID-19 information found online

Students perceived the information they had found on the internet as applicable to everyday life and usable for decision-making. In examining the difficulty levels in these, the median answer within both genders and all age groups was ‘Easy’ for all of the three items. There were no statistically significant differences between genders or age groups (Table 1).

**Table 1:** Higher education students’ self-perceived abilities of applicability and usability of the COVID-19 information found online, by gender and age. The numbers stand for: 1 = Very easy, 2 = Easy, 3 = Difficult, 4 = Very difficult

Demographics (*N* = 2341)	COVID-19 information found on the internet												
	Applicability for decision-making				Applicability to daily life				Usability to make decisions about health				A mean from the sum of the three self-perceived abilities
Gender	%	*Mdn*	*M*	*SD*	*%*	*Mdn*	*M*	*SD*	*%*	*Mdn*	*M*	*SD*	*M*
Female	65	2.00	1.954	0.647	57	2.00	1.901	0.569	65	2.00	1.96	0.652	1.938
Male	64	2.00	1.917	0.638	59	2.00	1.911	0.592	64	2.00	1.928	0.643	1.919
	*H(1) = *1.84, *p = *0.1739				*H(1) = *0.01, *p = *0.9016				*H(1) = *1.68, *p = *0.1946				Welch Two-Sample *t-*test *t = *0.76, *df = *998.42, *p = *0.4444
Age													
<22	66	2.00	1.972	0.659	54	2.00	1.904	0.541	63	2.00	1.928	0.632	1.933
22–24	63	2.00	1.926	0.629	58	2.00	1.892	0.583	65	2.00	1.955	0.647	1.924
25–29	65	2.00	1.96	0.648	57	2.00	1.915	0.573	64	2.00	1.94	0.645	1.939
30–34	66	2.00	1.917	0.657	61	2.00	1.905	0.605	69	2.00	2.016	0.693	1.946
	*H(3) = *2.98, *p = *0.394				*H(3) = *1.03, *p = *0.7925				*H(3) = *3.71, *p = *0.2935				ANOVA *df = *3, *MS = *0.4211, *F = *0.166, *p = *0.919

Notes: %, *Mdn*, *M* and *SD* represent percent, median, mean and standard deviation. *H*(*df*) = Kruskall–Wallis chi-square, *p* = *p*-value. Also, *t* = *t*-statistics, *df* = degrees of freedom, MS = mean squares and *F* = *F*-value.

### Online information search on COVID-19 and mental well-being

The mean of mental well-being scores among the students who searched for the COVID-19 information online was 52.85 (*SD = *18.09). Among those who did not search for information online, the score was *M *= 54.21 (*SD *= 18.45). No statistically significant difference in mental well-being was found between students who searched for information and those who did not (see the ANOVA results in Table 2).

**Table 2:** ANOVA for the higher education students’ mental well-being according to COVID-19 online information search (no vs. yes), by gender and age

The dependent variable: mental well-being	*df*	*MS*	*F*	*p*
*Independent variables*				
Gender	1	2537.2	7.721	0.005
Search for COVID-19 information from the internet	1	661.9	2.014	0.156
Search for COVID-19 information from the internet * gender	1	24.7	0.075	0.784
Age	3	299.4	0.908	0.436
Search for COVID-19 information from the internet	1	661.9	2.008	0.157
Search for COVID-19 information from the internet * age	3	70.4	0.214	0.887
*Interaction between gender, age and search for the COVID-19 information from the internet*				
Gender	1	2497.2	7.591	0.005
Age	3	299.4	0.910	0.435
Search for COVID-19 information from the internet	1	661.9	2.012	0.156
Search for COVID-19 information from the internet * gender	1	33.1	0.101	0.751
Search for COVID-19 information from the internet * age	3	58.7	0.178	0.911

Notes: *df* = degrees of freedom, *MS* = mean squares, *F* = F-value, *p* = *p*-value.

There was a gender difference in mental well-being scores, *F*(1) = 7.721, *p = *0.005 (see Table 2). The mean well-being score among male students (*M *= 54.74, *SD *= 18.38) was higher compared with female students (*M *= 52.48, *SD *= 18.03). Among the different age groups, the means of the well-being scores were quite similar. The score was highest among the 30- to 34-year-old (*M *= 54.14, *SD *= 18.74), followed by the age groups of 22–24 (*M *= 53.37, *SD *= 18.17), < 22 (*M *= 52.85, *SD *= 17.15) and 25–29 (*M *= 52.42, *SD *= 18.52). The interaction between the COVID-19 information searched online and gender/age was not significant.

### Ability to determine the relevance of online information on COVID-19 and mental well-being

The ability to determine the relevance of online information about COVID-19 was associated with students’ mental well-being in all of the three items. On the rating scales ‘difficult’ and ‘very difficult’, the scores demonstrated low-mental well-being (a cut-off score of < 50) for all of the items (Table 3).

**Table 3:** Higher education students’ mental well-being scores on self-perceived ability to determine the relevance of COVID-19-related online information for daily decision-making and for health

Determining the relevance of COVID-19 health information	Mental well-being scores on self-perceived ability								
	Very easy		Easy		Difficult		Very difficult		
	*M*	*SD*	*M*	*SD*	*M*	*SD*	*M*	SD	*p*
Applicability for decision-making	56.80	18.92	52.95	17.32	47.43	17.50	38.25	28.80	*p* < 0.001
Applicability to daily life	57.33	18.78	52.51	17.50	46.39	17.67	43.69	24.95	*p* < 0.001
Usability to make decisions about health	56.02	19.22	53.10	17.52	47.63	17.24	45.66	19.81	*p* < 0.001

Notes: *M* and *SD* represent mean and standard deviation, *p* = *p*-value.

According to the ANOVA, gender was statistically significantly associated with well-being scores (Table 4): applicability for decision-making, *F*(1) = 6.010, *p *= 0.014; applicability to daily life, *F*(1) = 7.082, *p *= 0.007; and usability to make decisions about health, *F*(1) = 5.858, *p *= 0.015. The interactions between gender and the three items were not statistically significant. Age was not statistically significantly associated with mental well-being scores for any of the items of self-perceived abilities of the COVID-19 information found on the internet (Table 4).

**Table 4:** ANOVA for higher education students’ mental well-being according to the self-perceived abilities of applicability and usability of the COVID-19 information found on the internet, by gender and age

The dependent variable: mental well-being	*df*	*MS*	*F*	*p*
*Independent variables*				
Gender	1	1908	6.010	0.014
Applicability for decision-making	3	7473	23.537	<0.001
Applicability for decision-making * gender	3	86	0.272	0.845
Gender	1	2250	7.082	0.007
Applicability for daily life	3	7026	22.114	<0.001
Applicability for daily life * gender	3	248	0.780	0.505
Gender	1	1876	5.858	0.015
Usability to make decision about health	3	5332	16.650	<0.001
Usability to make decision about health * gender	3	155	0.484	0.693
*Independent variables*				
Age	3	255	0.805	0.491
Applicability for decision-making	3	7473	23.539	<0.001
Applicability for decision-making * age group	9	448	1.410	0.178
Age	3	279	0.878	0.451
Applicability to daily life	3	7026	22.125	<0.001
Applicability to daily life * age	9	564	1.776	0.067
Age	3	407	1.266	0.285
Usability to make decisions about health	3	5332	16.588	<0.001
Usability to make decisions about health * age group	9	109	0.388	0.963

Notes: *df* = degrees of freedom, *MS* = mean squares, *F* = F-value, *p* = *p*-value.

The means of the mental well-being scores among females and males were highest in the ‘very easy’ category among all items. Males had higher scores compared with females. The mean scores in the ‘very easy’ category for the first item, applicability for decision-making, were 58.44 (*SD *= 18.47) for males and 56.23 (*SD *= 19.07) for females. For the second item, applicability to daily life, the mean scores were 57.76 (*SD *= 19.56) for males and 57.18 (*SD = *18.54) for females. For the third item, usability to make decisions about health, the mean score among males was 56.93 (*SD *= 19.24), and for females it was 55.72 (*SD *= 19.23).

Among all age groups, the means well-being scores were the highest in the ‘very easy’ category for all items. The score was highest in the ‘very easy’ category in item applicability for decision-making among 30- to 34-year-old (*M *= 60.96, *SD *= 18.66), followed by the age groups of 22–24 (*M *= 57.20, *SD *= 19.36), 25–29 (*M *= 55.39, *SD *= 19.08) and < 22 (*M *= 55.30, *SD *= 17.85). The score was also the highest in applicability to daily life among 30- to 34-year-old (*M *= 61.03, *SD *= 19.20), followed by the age groups of 22–24 (*M *= 57.81, *SD *= 18.86), < 22 (*M *= 56.13, *SD *= 16.96) and 25–29 (*M *= 55.76, *SD *= 19.43). In the usability to make decisions about health, the highest scores were among 30- to 34-year-old (*M *= 58.12, *SD *= 19.96) followed by 22–24 (*M *= 57.16, *SD *= 18.27), < 22 (*M *= 55.38, *SD *= 18.26) and 25–29 (*M *= 54.41, *SD *= 20.44).

## DISCUSSION

The findings of this study indicate that a majority of Finnish HE students (over 80%) had used the internet to search for the COVID-19 information during the pandemic and the associated lockdown of the HE institutions in May 2020. It is noteworthy that small gender and age differences existed: females searched for health information more frequently compared with males. As students’ age increased, a higher percentage of them searched for information online. In addition, with regard to determining the relevance of information for everyday health decision-making, students perceived it as easy.

Codirectional results have been reported in another Nordic country (Denmark) within the COVID-HL Network, which found that most HE students believed that they can apply information about coronavirus in their daily lives and that it is applicable to them (Bak *et al.*, 2022). This demonstrates that students are seeking online health information because the internet provides abundant, easy and quickly retrieved information, as has been indicated in previous studies (Wang *et al.*, 2020; Rouvinen *et al.*, 2021). However, even though an individual may experience OHIS as easy, they might be focusing on reading information only from certain online environments that serves their existing beliefs (Meppelink *et al.*, 2019). Nevertheless, OHIS and the individual ability to find and use relevant information is a necessary skill among students, because they are in a life phase when health-related decisions are usually made independently (Wang *et al.*, 2020; Zhang *et al.*, 2021).

It should be noted that at the time of the survey, the mass media and other information channels that utilise the internet to disseminate information had already provided information intensively on COVID-19 for several months (Finnish Government, 2022). It was already known that the symptoms of COVID-19 ranged from very mild (fever and respiratory symptoms) to severe (pneumonia, severe acute respiratory syndrome and kidney failure; WHO, 2020). Hence, there is a possibility that students who had concerns about COVID-19 had already sought and obtained information online and the availability of information had already been apparent (Le *et al.*, 2020).

Females used the internet more than males to search for the COVID-19 information. The existing literature indicates that females, in general, have a higher frequency of searching online health information because of their emotional and social characteristics. For example, females might be more socially engaged compared with males and try to find emotional support in online communities within social networking sites such as social media (Baumann *et al.*, 2017; Hassan and Masoud, 2020). In addition, females are more likely to seek health information online compared with males when they experience poor health (Nikoloudakis *et al.*, 2018). The present results indicate that, among the students who used the internet for COVID-19 information-searching, females reported having slightly poorer mental well-being scores compared with males. This might be because females and males tend to use different online sources for health information searching. Females tend to use health forums, online video sharing platforms, social media, blogs and search engines more frequently than males, who tend to use apps and Wikipedia and other web-based encyclopaedias more (Bauman *et al.*, 2017; Dadaczynski *et al.*, 2021a).

According to the Finnish HE students’ health survey from 2016, female students reported a poorer health status, more health symptoms and more mental health problems compared with males (Kunttu *et al.*, 2017). More specifically, females tend to suffer from depression, anxiety, eating or somatoform disorders, whereas males are more prone to substance use and antisocial disorders (Huang *et al.*, 2018; Wenjuan *et al.*, 2020). It is worth acknowledging that the mean WHO-5 well-being scores of both genders in this study were a little over 50, which indicates sufficient well-being. However, this also suggests reduced well-being for several students because the cut-off point on the WHO-5 well-being scale is < 50, which indicates low well-being (Topp *et al.*, 2015). In another COVID-HL study conducted in Germany, close to 40% of the students reported lower well-being (Dadaczynski *et al.*, 2021b). The COVID-19 pandemic, as well as its lockdowns and social isolation effects on HE students’ mental well-being, have recently been widely studied, and the evidence indicates that stress, anxiety and depressive thoughts increased because of the COVID-19 pandemic (de Oliveira *et al.*, 2020; Sarasjärvi *et al.*, 2022).

The present findings show that students who reported difficulties applying health information retrieved online also had lower mental well-being compared with those who considered applying information ‘very easy’ and who had higher mental well-being scores. This indicates that there are positive associations with the individual’s ability to transfer information to everyday practice, as has also been recognised in other studies of OHIS behaviour (Lattie *et al.*, 2019; Capone *et al.*, 2020). It is, however, noteworthy that the well-being scores were lower when the items of the DHLI subscale ‘determining relevance’ were experienced as more difficult. It also demonstrates that even though DHL would be well developed, some students face individual difficulties in evaluating and using the information for their health (Dadaczynski *et al.*, 2021a; Patil *et al.*, 2021). This might be due to difficulties in assessing the trustworthiness of media information on COVID-19 and the associated health problems, as well as confusion on the massive amounts of information available (Dadaczynski *et al.*, 2021a). It also could be that students do not recognise the need for treatment but rather accept that mental health problems, such as depression and anxiety symptoms, are typical expressions of stress (Lattie *et al.*, 2019.) Several strategies and resources exist to strengthen mental health, for example, the REDFLAGS model provides guidance on recognising warning signs that students with mental health concerns might exhibit. The warning signs include eight behavioural indicators of general and common symptoms of mental health disorders in students. REDFLAGS is a tool intended for educators so that they can support and guide those in need to the students’ counselling centre or to other resources (Kalkbrenner, 2016).

Our results demonstrate that gender plays a part in students’ mental well-being and in using (or not using) the internet to search for the COVID-19 information. Gaining further understanding of gender differences in health-related internet use may help health communication specialists develop online health-related information sharing, for example, in general public forums and in exchanging medical information with health care professionals (Bauman *et al.*, 2017). To further understand students’ behaviour and intentions for online health-related information-seeking behaviour from the perspective of health communication, the PRISM model (Kahlor, 2010), as introduced earlier, offers variables for individual-level prediction. Notably, all factors apart from perceived knowledge are theorised to positively relate to information-seeking intention (Wang *et al.*, 2020). This could mean that, during COVID-19, students’ perceived knowledge insufficiency and risk perception, as well as their affective responses, could be key intentions to using the internet for health-related information. In particular, the fear of inadequacy and the rapidly changing nature of health information have been concerns linked to information searched on the internet among students (Patil *et al.*, 2021). After the intention, however, the possibility to access and use health information predicts health-related behaviours as increased cognitive knowledge in health can lead to behavioural outcomes (Lee *et al.*, 2021). In addition, adequate health literacy skills are required to make decisions about health and health behaviour (Manganello *et al.*, 2017). As a consequence, within health communication, the provision of easily available, clear and consistent health-related information is essential when populations with lower health literacy are taken into consideration.

### Limitations

As a limitation of this study, it should be noted that students’ OHIS on other health issues or risks apart from COVID-19 may differ. In addition, this study did not directly focus on the concept of health literacy among the students but instead on OHIS behaviour that benefits from health literacy skills and thus cannot be directly compared with health literacy studies. It is also possible that there might be students who think they are able to properly find and apply health information online but who are using unreliable sources. The depth of individual ability, and the ability to process and apply health information, were not evaluated in this study, and require more examination in the future. Another limitation is that the study recruited students online only, through universities’ own information channels and thus may not have reached students who do not use these channels or who do not use them frequently. However, the percentage of those might be considered low given the fact that most students are familiar with the channels used. A final limitation is that our study is based on cross-sectional data, and therefore, no causality statements can be derived.

## CONCLUSION

As a conclusion, the results demonstrated that most of the HE students used the internet to search for the COVID-19 information and perceived finding the information to be easy. The well-being scores, however, were lower when determining the relevance of the information was experienced as more difficult. This indicates that supporting students’ individual ability to understand and use health information retrieved online could contribute to better personal mental well-being. It is also worth noting that educating students about the importance of using reliable internet sources for health is equally important. DHL is an important factor to consider when examining students’ online health information-seeking behaviour. Overall, this study provides insights into the promotion of HE students’ health-related decision-making and behaviour. It also demonstrates the popularity of the internet as a channel for health information during COVID-19. As the pandemic has continued after the collection of these data, more research on students’ online health information-seeking behaviour and well-being is needed.

## Supplementary Material

daad143_suppl_Supplementary_Files_S1
