# Supplementary material for: Online health information-seeking behaviour and mental well-being among Finnish higher education students during COVID-19
Source: Health Promot Int. 2023 Nov 7;38(6):daad143. doi: 10.1093/heapro/daad143 (PMC10635413; doi:10.1093/heapro/daad143)
Supplement: daad143_suppl_Supplementary_Files_S1 [file daad143_suppl_supplementary_files_s1.docx]

**Supplementary File S1.** Higher education students’ online search for COVID-19 information by gender and age

| Demographics | Internet use to search COVID-19 information | | |
| --- | --- | --- | --- |
|  | **Yes**  **n (%)** | **No**  **n (%)** | **Total** |
| Gender |  |  |  |
| *Female* | 1789 (86%) | 300 (14%) | 2089 |
| *Male* | 597 (82%) | 132 (18%) | 729 |
| ¹¹p= 0.018  Cramér's V effect size= 0.04 |  |  |  |
| Age group |  |  |  |
| *<22* | 467 (80%) | 117 (20%) | 584 |
| *22-24* | 856 (85%) | 148 (15%) | 1004 |
| *25-29* | 742 (86%) | 120 (14%) | 862 |
| *30-34* | 321 (87%) | 47 (13%) | 368 |
| ¹¹¹p=0.004  Effect size coefficient= 0.06 |  |  |  |

*p*

¹¹ *p* = Pearson’s statistical significance for the gender difference

¹¹¹ *p* = Pearson’s statistical significance for the age difference
